# Developing a rehabilitation intervention for eating and drinking difficulties following stroke through co-design stakeholder workshops

**DOI:** 10.1186/s12913-026-14977-2

**Published:** 2026-06-23

**Authors:** Natalie Jones, Susan Mawson, Avril Drummond, Lily Booth, Polly Sedman, Erin Horner, Marge Allen, Alicia O’Cathain

**Affiliations:** 1 ScHARR, Division of Medicine and Population Health, University of Sheffield, 3rd Floor, 30 Regents Court, Sheffield, S1 4DA UK; 2 Sheffield Teaching Hospitals NHS Foundation Trust, Norfolk Park Road, Beachhill, Sheffield, S2 3QE UK; 3 School of Sport, Exercise and Health Sciences, Loughborough University, Epinal Way, Loughborough, Leicestershire, LE113TU UK; 4 The Rotherham NHS Foundation Trust Hospital, Moorgate Road, Rotherham, S60 2UD UK; 5 Doncaster and Bassetlaw Hospitals, Armthorpe Road, Doncaster, DN2 5LT UK; 6 Primary Care Doncaster Limited, Oak Tree Lodge, Tickhill Road Site, Balby, DN4 8QN Doncaster, UK

**Keywords:** Stroke, Patient and public, Experience-based co-design, Intervention development, Eating and drinking, Participatory research

## Abstract

**Background:**

Eating and drinking difficulties are common after stroke and have profound physical, psychological, and social consequences. Although mealtime group activities are commonly used in rehabilitation settings to support recovery, these approaches are rarely standardised, systematically developed, or evidence-informed, and there is limited research evaluating structured group-based mealtime interventions in stroke care. Co-produced, contextually grounded interventions may improve engagement and clinical relevance. This study aimed to co-design a breakfast group intervention to address eating and drinking difficulties for people after stroke in hospital settings.

**Methods:**

This intervention development study was conducted across three secondary care hospital stroke wards. Ten online workshops were held with a 15-member Stakeholder Intervention Development Group comprising stroke survivors, informal carers, and multidisciplinary healthcare professionals. An iterative co-design process was used, informed by prior systematic review findings, observational work, interviews, and trigger films exploring lived experiences. Intervention development combined Hawkins’ framework with Experience-Based Co-Design. Workshops involved structured co-design activities, group discussion, and reflection on personal experiences. Data from workshops were synthesised collaboratively to refine intervention content and delivery.

**Results:**

Stakeholders co-produced the Breakfast Group Intervention for Stroke Rehabilitation (BISTRo) and an accompanying implementation toolkit. Five core components were identified: (1) multidisciplinary participation, (2) physical rehabilitation, (3) food preparation and choice, (4) peer support, and (5) psychological well-being. The intervention was tailored for delivery on hospital stroke wards, with adaptations for infection control procedures and local contextual variation. Online participatory methods enabled sustained engagement and inclusive contribution from diverse stakeholders.

**Conclusions:**

Using Experience-Based Co-Design within Hawkins’ framework supported the inclusive and iterative development of a complex rehabilitation intervention. This co-produced approach generated a contextually grounded breakfast group model suitable for feasibility testing and future evaluation. The findings highlight the value of stakeholder partnership in designing health service interventions addressing post-stroke eating and drinking difficulties.

**Trial registration:**

The study was registered on Clinical Trials.gov ID NCT05102812 on the 17th of February 2022.

**Supplementary Information:**

The online version contains supplementary material available at https://doi.org/10.1186/s12913-026-14977-2.

## Introduction and background

Eating and drinking difficulties are highly prevalent following stroke. Dysphagia is a common post-stroke complication with reported prevalence estimates ranging from 18 to 81% depending on the stage of recovery, stroke severity, and assessment method used. A recent systematic review and meta-analysis reported an overall prevalence of 43.6%-46.6%, depending on stroke type [1, 2]. Dysphagia increases the risk of pneumonia [3], malnutrition [4] and reduced quality of life [5]. Such complications have been linked to increased mortality and longer hospital admissions [3, 6]. In addition, motor impairments commonly seen after stroke can affect arm function, postural control, and mobility, restricting the ability to feed oneself safely and independently [7].

While the physical consequences of eating and drinking difficulties after stroke are well recognised, the psychological and social impacts are less well known [8]. Stroke survivors report shame and humiliation [9], bewilderment, dismay, and despair [10] and a loss of pleasure associated with reduced social eating [11], reduced enjoyment, social isolation, and diminished confidence [2, 11, 12]. These experiences can further hinder recovery and participation.

Despite this, rehabilitation practice often prioritises physical function, with limited attention to psychosocial and behavioural dimensions of eating and drinking [13, 14]. Rehabilitation of eating and drinking is recognised as essential to support adaptation, compensation, risk reduction, and regain participation, consistent with national guidance [15]. Mealtime groups are increasingly used within stroke rehabilitation to enhance intensity and facilitate peer support [15]. Group-based mealtime rehabilitation approaches are used within some stroke rehabilitation services to enhance therapy intensity and facilitate peer support; however, these approaches are rarely standardised or systematically developed as multidisciplinary interventions, and there is limited published research evaluating structured group-based mealtime interventions in stroke care. Together, these factors highlight the need for multidimensional interventions that address the physical, psychological, and social complexity of eating and drinking difficulties after stroke.

The Breakfast Group Intervention for Stroke Rehabilitation (BISTRo) was developed in response to this gap, with the aim of restoring enjoyment, autonomy and social connection in eating and drinking while supporting safe and evidence-based rehabilitation practices. Developing interventions of this nature requires methods that meaningfully engage those who will use, deliver and be affected by them [16]. To ensure the intervention was conceptually robust, contextually relevant, and acceptable to those who would use it, BISTRo was developed through a structured intervention-development process underpinned by recognised frameworks and participatory co-design. This paper describes the development of BISTRo, including its theoretical foundations, stakeholder involvement, and the workshop processes through which the intervention was constructed.

### Ethical considerations

All procedures performed in this study involving human participants were carried out in accordance with the Declaration of Helsinki and the UK Good Clinical Practice (GCP) guidelines. Ethical approval was obtained from the appropriate NHS Ethics Board. Ethical approval for the study was granted by the Northwest-Haydock NHS Research Ethics Committee on 5 January 2021 (REC 21/NW/0313). The study was registered on Clinical Trials.gov ID NCT05102812. All participants received a detailed information sheet describing the study purpose, procedures, confidentiality arrangements, and the voluntary nature of participation. Written informed consent was obtained either in person or electronically, according to participant preference and in line with COVID-19 restrictions [17].

To support informed consent for stroke survivors with communication or cognitive impairments, participant information materials were designed using accessible language and aphasia-friendly formatting where appropriate. Potential participants were given sufficient time to review study information, discuss participation with family members or clinicians if desired, and ask questions before providing consent. Researchers used supported communication strategies, including simplified language, repetition, visual prompts, and checking understanding throughout the consent process. In accordance with Health Research Authority guidance, participants received a modest voucher and a certificate of contribution upon completion [18]. Workshop sessions were recorded only with explicit consent, and participants were free to keep cameras switched off or to contribute verbally without being recorded if they preferred.

## Methods

This study aimed to co-produce the content of a structured breakfast group intervention to support eating and drinking rehabilitation for people after stroke in hospital settings.

### Setting

The study was conducted across three secondary care NHS hospital stroke wards in England. All co-design workshops were delivered online due to COVID-19 restrictions. This was a qualitative intervention development study; therefore, no statistical comparisons or power calculations were undertaken.

### Study design

A range of frameworks exist to guide complex intervention development [19], and such approaches are recommended to support methodological rigour and transparent reporting [20, 21]. Hawkins et al. [16] propose a three-stage framework comprising: (i) evidence review and stakeholder consultation, (ii) co-production of intervention content, and (iii) prototyping and refinement. Stage 1 has been reported elsewhere [22]. Stage 2, presented in this paper, focused on co-producing the content of the intervention [23].

Co-design approaches are increasingly used in healthcare research to support the development of interventions that are acceptable, feasible, and tailored to local context [24]. As Hawkins provides limited procedural guidance for co-design, principles from Experience-Based Co-Design (EBCD) were incorporated [25, 26]. EBCD brings patients, carers, and healthcare professionals together to collaboratively design services using experiential evidence, including narrative triggers such as lived-experience videos [21, 27, 28].

Stage 2 centred on stakeholder collaboration to design and refine the intervention. Stakeholders included stroke survivors, informal carers, and multidisciplinary rehabilitation professionals [21, 22, 29]. Meaningful involvement is recognised as key to improving relevance, acceptability, sustainability and supporting translation of research evidence and theory into practical intervention design [21, 24, 30]. To achieve this, three steps were undertaken: (i) establishment of a Stakeholder Intervention Development Group; (ii) delivery of a series of participatory workshops to co-design the intervention and implementation toolkit; and (iii) synthesis of outputs into a prototype suitable for feasibility testing.

### Data analysis

The study was informed by a pragmatic and interpretive qualitative approach, recognising that knowledge was co-constructed through interaction between stakeholders, researchers, and the wider rehabilitation context. Ontologically, the study assumed that experiences of eating and drinking difficulties after stroke are socially and contextually situated. Epistemologically, knowledge was understood as co-constructed through interaction between stakeholders, researchers, and the wider rehabilitation context. This positioning aligned with Experience-Based Co-Design principles and supported collaborative interpretation of experiential and empirical evidence during intervention development.

Workshop data were analysed using Framework Analysis [31] a systematic approach to qualitative data analysis commonly used in applied health research to support comparison across participant groups, data sources, and timepoints. To support collaborative interpretation and co-design, findings from earlier qualitative interviews [22], participant observations, and literature review data [14] were synthesised into thematic summaries and visual mind maps illustrating relationships between themes and subthemes. These materials were circulated to stakeholders in advance of workshops to support familiarisation and reflection. During workshops, participants discussed their own experiences in relation to the presented findings, challenged or expanded interpretations, and collaboratively identified priorities for intervention development. This iterative interpretive process enabled experiential knowledge and empirical evidence to inform the evolving intervention design.

Workshop recordings, field notes, chat transcripts, and workshop artefacts were reviewed after each session by the lead researcher (NJ). Data were coded inductively and organised using framework analysis [31]. Initial coding focused on concepts relating to intervention content, delivery, contextual barriers and facilitators, and stakeholder priorities. Codes were iteratively grouped into categories and broader themes, which were refined through comparison across workshops and stakeholder groups. Framework matrices were used to document emerging interpretations, design decisions, and changes to the intervention over time. Synthesised findings from each workshop informed the content and structure of subsequent workshops, enabling iterative refinement of the intervention prototype and implementation toolkit.

The study has been reported using the CReDECI 2 checklist for complex intervention development [32] (Supplementary Table 2). We followed the reporting guidance for intervention development studies [33] and the GRIPP-2 guidance [29] (Supplementary Table 3) for reporting patients and public involvement in health care research. A table summarising how the CReDECI 2, GRIPP 2 and Duncan et al. [33], reporting guidelines (Supplementary Table 4) were met is provided in the supplementary information.

### Stakeholder intervention development group (SIDG)

#### Participants and recruitment

The SIDG included healthcare professionals, stroke survivors, and informal carers recruited through NHS stroke services and public involvement networks in one region. Recruitment materials, including posters and participant information sheets, were disseminated through the regional Stroke Integrated Delivery Network and a local hospital stroke survivor patient and public involvement group.

Eligibility criteria were deliberately broad to promote diversity of perspectives. Healthcare professionals from secondary care organisations were eligible if they were currently or recently working in stroke rehabilitation and included occupational therapists, speech and language therapists, physiotherapists, dietitians, nurses, and support staff. Stroke survivors and informal carers were eligible if they had experienced eating or drinking difficulties following a stroke and could contribute to online discussions.

Purposive sampling was used to achieve variation in professional role, seniority, experience, and demographic characteristics [30, 34]. We initially sought to recruit 10 stakeholders. Unexpectedly high levels of interest, combined with the likelihood of attrition due to COVID-19 pressures, led to a decision to over-recruit stakeholders.

#### Codesign workshops

Ten online stakeholder workshops were conducted between June and November 2022. Workshops were undertaken as a research method, so they are reported transparently [24]. Workshops took place during the COVID-19 pandemic, when face-to-face engagement was restricted. As a result, all workshops were conducted online using Microsoft Teams and NHS-approved secure account. While this presented challenges, including variable access to technology and the difficulty of building rapport remotely, it also broadened participation and demonstrated the feasibility of virtual co-production in rehabilitation research [17, 25, 35].

**Fig. 1:**
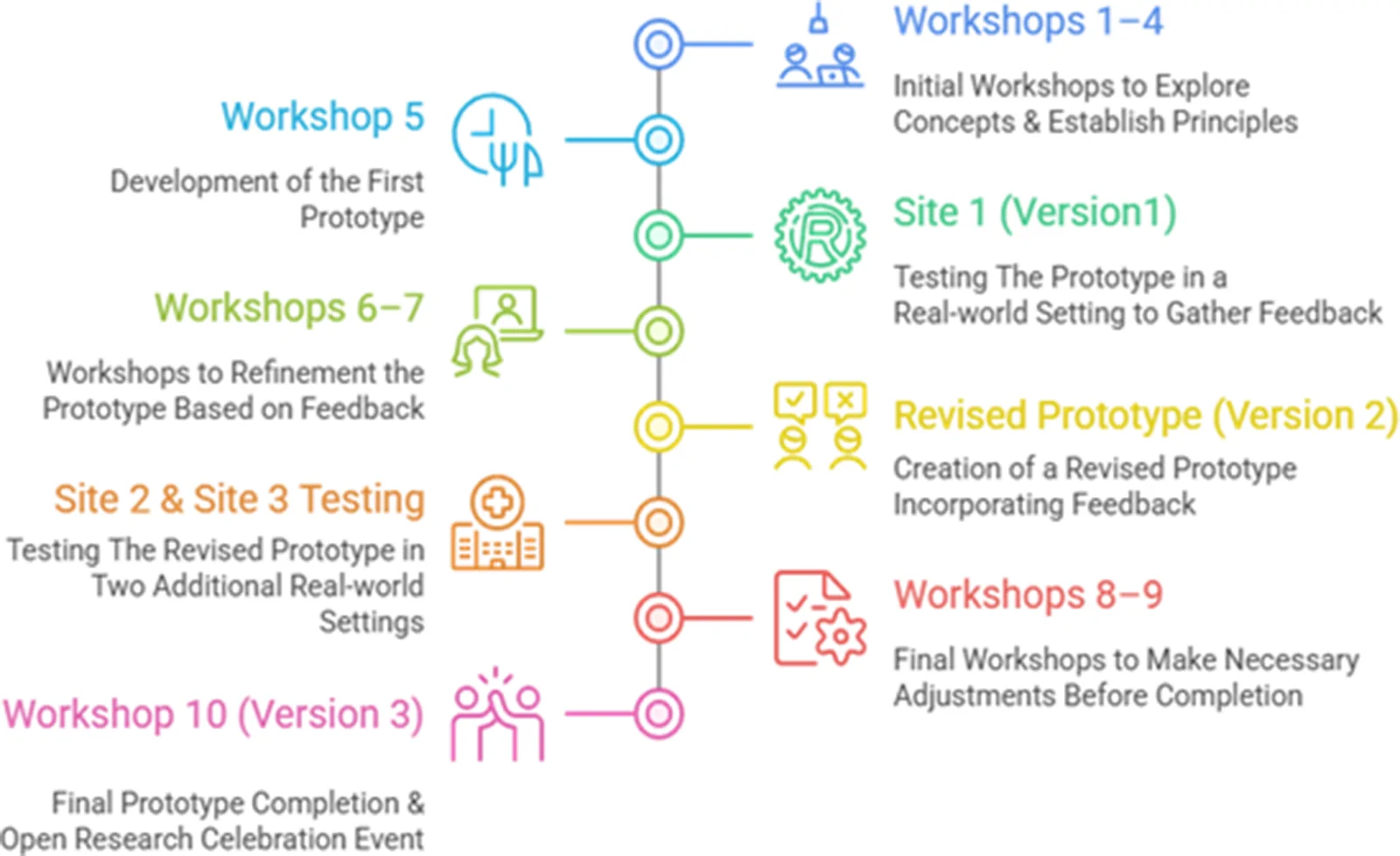
Overview of the iterative co-design process. Figure 1 Overview of the iterative co-design and prototyping process across the ten stakeholder workshops conducted over a four-month period. Workshops 1–4 focused on the exploration of concepts and establishing principles. Prototype Version 1 was developed in Workshop 5 and tested in Site 1 prior to Workshop 6. Workshops 6–7 focused on refinement of the prototype based on feedback. The revised prototype (Version 2) was subsequently tested in Sites 2 and 3 prior to final refinement during Workshops 8–9. Workshop 10 focused on completion of the final prototype and dissemination activities

We designed the workshops to enable progressive co-design of the intervention through structured discussion, creative exercises, and reflection. Each session had a bespoke agenda circulated in advance and opportunities for participants to amend or add topics. The duration was one-hour which was reasonable to stroke survivors, carers and staff who were working under extreme pressure during the pandemic. The lead author (NJ), an occupational therapist who specialised in stroke rehabilitation, facilitated the workshops, supported by co-leads (EH, PS, LB), encouraging shared leadership and participatory decision-making consistent with EBCD principles [21, 36]. The theoretical basis drew on rehabilitation theory [37], psychosocial recovery [38, 39], neurorehabilitation theory [40], therapeutic meal activity, combined with Hawkins’ [16] structured approach and EBCD principles [21]. Figure 1. provides an overview of the iterative co-design process and prototyping timeline.

Workshops 1–4 focused on exploring learning from the evidence and stakeholder engagement from Stage 1 of Hawkins’s approach [16, 22]. Trigger videos, which showed patients, informal carers and health care professionals talking about what mattered to them about eating and drinking post stroke, were used in these sessions to prompt emotional engagement and discussion around lived experience [23] (Supplementary Videos 1). These early workshops also focused on establishing group values and identifying priorities for the intervention. Workshops 5–7 concentrated on prototyping different aspects of the intervention and giving feedback on them. Workshops 8–10 involved refining the intervention and celebrating the SIDG members’ achievements.

Creative techniques such as virtual whiteboards, virtual post-it notes, and mind mapping were used to capture ideas, while smaller group discussions enabled inclusion [18]. Each workshop concluded with a summary of decisions and agreed next steps, which were documented and circulated alongside an agenda for the following meeting.

Multiple sources of data were generated during the workshops, including verbatim transcripts of the meeting, field notes made by the facilitator, reflective memos, transcripts from the ‘chat’ function of the online meeting software, and artefacts such as mind maps and whiteboard outputs produced by the SIDG during workshops. This data provided a rich record of decision-making and creative processes.

#### Data synthesis and analysis

This approach enabled inductive identification of emerging concepts while facilitating comparison across stakeholder groups and timepoints, making it well suited to iterative co-design [41, 42]. A matrix-based framework was used to chart workshop discussions, design decisions, and reflections, supporting transparency, traceability of changes, and integration of findings into subsequent workshops. This approach aligned with the study’s participatory ethos by preserving stakeholder contributions and ensuring that the evolving intervention remained grounded in experiential data.

Initial coding focused on concepts related to the structure, content, and delivery of the emerging intervention. Synthesised findings from each workshop were fed forward into subsequent workshops, enabling iterative refinement and continuity across the co-design process [24]. By Workshop 5, an initial intervention prototype had been developed and tested in one hospital. Learning from this pilot was reviewed in Workshop 6, leading to further amendments. The revised prototype was subsequently tested in two additional hospitals, with findings reviewed in Workshops 8 and 9. Workshop 9 was dedicated to finalising the intervention prototype.

To enhance analytical rigour, the lead author applied a triangulation protocol [43], systematically comparing workshop outputs with findings from earlier study phases, including ethnographic fieldwork, interviews, and observational data. This ensured that the evolving intervention remained anchored in empirical evidence while reflecting stakeholder priorities and lived experiences.

### Patient and public involvement statement

Patients and members of the public (PPI) were involved from the earliest stages of the BISTRo study. Prior to submission of the fellowship application, a PPI group was convened to discuss the overall aims, relevance, and acceptability of the proposed research. The PPI group comprised stroke survivors with lived experience of stroke rehabilitation and eating or drinking difficulties, recruited through local stroke support networks and hospital-based patient involvement groups. They contributed specifically to the broader advisory and oversight aspects of the study, including study design, recruitment processes, participant materials, and dissemination planning. The PPI group was distinct from the Stakeholder Intervention Development Group (SIDG). The PPI group provided advisory input throughout study development and dissemination planning, whereas the SIDG was established specifically to undertake the structured co-design workshops described in Stage 2 of the Hawkins Framework [16].

The research questions and outcome measures were refined in collaboration with PPI contributors, ensuring that they reflected outcomes that mattered to patients and families rather than solely clinical or academic priorities. Discussions focused on lived experience, acceptability of change, and what “success” would look like from a patient and family perspective. This input informed both the primary and secondary outcomes, as well as decisions about how these outcomes would be measured and presented.

Patients and public contributors were actively involved in study design. They reviewed and commented on the protocol, ethics application, and participant-facing materials, including information sheets and consent forms, to ensure that language was clear, sensitive, and non-coercive. They also provided advisory feedback on the planned intervention development process and interview topic guide, helping ensure that the overall approach remained meaningful, acceptable, and feasible from a patient and family perspective. The detailed co-design of the intervention itself was subsequently undertaken through the separate Stakeholder Intervention Development Group (SIDG) described in Stage 2 of the Hawkins Framework.

The PPI group was consulted about strategies for recruitment and the conduct of the study. They advised on appropriate routes and settings for identifying potential participants and how best to introduce the study to minimise burden and maximise inclusivity. They were also asked to comment on the burden of participation, including the time commitment required, number of contacts, and emotional demands. Their feedback led to adjustments in how and when data collection occurred and to the provision of additional support and flexible scheduling.

Public contributors were, and will continue to be, involved in the dissemination of findings. They advised on audiences, timing, and preferred formats for sharing results, including lay summaries for participants and wider patient communities, and potential routes such as charities, support groups, and social media. They will also co-produce accessible summaries of the findings to ensure that outputs are understandable and relevant to non-academic audiences. PPI contributors received regular feedback on how their input shaped the study and were offered appropriate reimbursement for their time and expertise in line with NIHR guidance.

## Results

Fifteen individuals consented to participate in the Stakeholder Intervention Development Group (SIDG), comprising healthcare professionals, stroke survivors, and an informal carer involved in the co-design workshops (thirteen women and two men), comprising four occupational therapists, four speech and language therapists, two dietitians, one nurse, one psychology assistant, two stroke survivors, and one informal carer. All participants self-identified as White British except two, a speech and language therapist and an occupational therapist, who both identified as White Irish (Table 1). Ten participatory workshops were delivered between June and November 2022. Attendance across the ten workshops was consistently strong, with between 9 and 15 SIDG members present at each session. At least nine stakeholders attended all workshops, and several remained engaged throughout the full co-design period, reflecting sustained commitment despite the demands of clinical work and the challenges of online participation. While attendance fluctuated slightly due to leave, shift patterns, and service pressures, the overall continuity of membership enabled iterative refinement of ideas and ensured that decisions were informed by a stable core group of contributors.

**Table 1:** Stakeholder characteristics

Stakeholder Group	Gender (*n*)	Total (*n*)
Occupational Therapists	Female (4)	4
Speech and Language Therapists	Female (3), Male (1)	4
Dietitians	Female (2)	2
Stroke Survivors	Female (1), Male (1)	2
Informal Carer	Female (1)	1
Nurse	Male (1)	1
Psychology Assistant	Female (1)	1
Total Participants	—	**15**

Characteristics of Stakeholder Intervention Development Group participants across professional and lived-experience roles

## The intervention

The Breakfast Group Intervention for Stroke Rehabilitation (BISTRo) is a structured, multidisciplinary group rehabilitation session for inpatients experiencing eating and drinking difficulties following stroke. It combines therapeutic eating and drinking, social engagement, and practical skill-building within a normalised dining environment. Group sessions (lasting 45–60 min, five days a week over two weeks) provide opportunities for patients to practice safe eating and drinking, rebuild upper limb function, work on posture and mobility and participate in peer interaction to enhance confidence and psychosocial well-being.

The intervention is facilitated primarily by occupational therapists, physiotherapists, dietitians, speech and language therapists, with input from nursing staff, therapy assistants, support workers, and, in one hospital, a psychology assistant as required. It is designed to integrate seamlessly into existing ward routines while promoting dignity, autonomy, and enjoyment at mealtimes.

The materials in the implementation ‘toolkit’ include: (i) an intervention training manual for health care professionals; (ii) a patient booklet where they and family can document their eating and drinking preferences, goals, a daily log, attendance at sessions, and discharge information; (iii) environmental checklists for how to set up the room and create the welcoming ambience (iv) communication tools (stickers, visual cues, staff prompts, name plates with diary specifications); (v) aphasia-friendly menus; and (vi) staff engagement resources such as conversational guidance for aphasic patients and conversational topic prompts. To ensure comprehensive and transparent reporting, the intervention is described in line with the Template for Intervention Description and Replication (TIDieR) checklist and guide [44] see supplementary information (Supplementary Table 1).

### Workshop 1: Establishing shared principles

Ground rules were co-developed to promote equity, inclusivity, and respectful dialogue, informed by the National Institute for Health Research co-production guidance [45]. An icebreaker activity using personal food stories fostered rapport. A trigger video portraying lived experiences of eating and drinking after stroke stimulated discussion about social connection, dignity, and enjoyment (Supplementary Videos 1). Key decisions included adopting the ground rules, establishing a ‘living’ considerations table, using the trigger video in later sessions, and documenting decisions transparently through summary tables and consideration logs.

### Workshop 2: Interpreting lived experience data

Findings from ethnographic observations, interviews, and the trigger video were presented. Discussion highlighted portion size, hunger, and communication barriers. Agreed actions included developing site-specific aphasia-friendly menus, incorporating dietary preferences into assessment, creating a COVID-safe standard operating procedure, and emphasising pleasure, autonomy, and person-centred choice within the intervention.

### Workshop 3: Defining core components and logic model

Participants brainstormed intervention components and refined a draft logic model, reframing ‘mastery’ as ‘confidence and participation.’ The group agreed that environmental design, multidisciplinary facilitation, and peer interaction should work synergistically to support safe eating, independence, and psychological well-being. Decisions included developing environmental guidelines, personalised communication aids, mechanisms for indirect carer input, and finalising a logic model to articulate assumptions, mechanisms, and intended outcomes.

### Workshop 4: Strengthening communication, engagement, and safety

Communication aids were finalised, including aphasia-friendly menus, dietary labelling, and visual cues. Plans were made for wider staff engagement to support implementation. COVID-19 contingency plans were drafted to allow safe intervention delivery under different infection-control restrictions. A patient-held booklet was also created to record goals, preferences, and progress and to support motivation and engagement.

### Workshop 5: Prototyping (Version 1)

Prototype materials and a draft training manual were reviewed and completed. Stakeholders recommended visual prompts to identify participants ready for the group (e.g. reusable stickers and ‘First-Up Boards’). Five core organising components were agreed: multidisciplinary facilitation; therapeutic physical activity related to eating and drinking; supported food preparation and personalised choice; peer interaction; and attention to psychological well-being. The decision was made to produce a single integrated patient–family booklet, and the training manual was refined to improve clarity before testing in site 1.

### Workshop 6: Prototype finalisation (Version 2)

Feedback from testing in site 1 emphasised accessibility, dignity, and independence. Eating and drinking aids such as non-slip mats and plate guards were found to be invaluable, and napkins were selected over plastic aprons, affirming the stakeholder discussion about dignity. Version 2 of the intervention prototype was commenced, alongside slight changes to the staff training manual and environmental setup checklists (Supplementary Fig. 1 Environmental Layout). Ongoing updates to infection-control guidance were incorporated to ensure alignment with organisational policies.

### Workshop 7: Refinement of Version 2

Findings from prototype testing in one hospital were discussed further and stakeholders recommended improvements to room layout for infection-control spacing, additional preparation stations (Supplementary Fig. 2 Food Preparation Station), and a redesigned the patient booklet with more writing space, integrated goals, and a sticker section to record attendance. Decisions included revising the booklet to support goal-setting, and encouraging patient-led food choices through including a section in the patient assessment to capture these before the intervention starts (Supplementary Fig. 3 Food Preferences).

### Workshop 8: Refinement following testing in Site 2

Findings from a second site test highlighted further patient booklet improvements, including additional space for the daily log (Supplementary Fig. 4 Daily Log), and enhancing the advice for home section. Treatment-plan sections were also simplified. An illustrator joined to discuss booklet images, with stakeholders emphasising representation of varied body shapes, cultures, and skin tones. Decisions included updating the training manual to recommend the groups have a consistent staff facilitator and adding prompts encouraging families to bring familiar food items to enhance enjoyment and cultural relevance.

### Workshop 9: Consolidation following testing in Site 3

Feedback from testing in site 3 and across all three hospital sites was reviewed. Although conversation-prompt tools for the social aspect had not been required in practice, stakeholders recommended including them as optional resources. Booklet illustrations were finalised with the illustrator following debate about whether to include weight-recording; a reflective page on diet concerns replaced weight monitoring (Supplementary Fig. 5 Healthy Eating). Core and adaptable components were agreed for fidelity and future feasibility testing.

### Workshop 10: Dissemination and celebration

A two-hour in-person open-research event simulated the breakfast-group environment, showcasing intervention materials, the training manual, posters (Supplementary Fig. 6 Codesign Poster), images, and patient participant quotations. Presentations were delivered by clinicians, stroke survivors, informal carers, and NHS leaders. A filmmaker documented the event, and key participants shared reflections; the final film and a poem written and read by a stroke survivor were presented (Supplementary Fig. 7 Poem). Feedback was invited via written reflections, reinforcing transparency, dialogue, and shared ownership. An exhibition area included patient booklet examples, photographs of the intervention in action, quotes, and illustrations (Supplementary Fig. 8).

## Discussion

This study reports the participatory development of the Breakfast Group Intervention for Stroke Rehabilitation (BISTRo), using Stage 2 of Hawkins’ intervention-development framework [16] integrated with Experience-Based Co-Design [21]. Across ten online workshops, stroke survivors, informal carers, and multidisciplinary professionals collaboratively designed and refined a rehabilitation intervention addressing the physical, social, and psychological dimensions of eating and drinking after stroke (Photograph of the group in action Supplementary Fig. 4). The process resulted in co-produced prototype materials ready for feasibility and acceptability testing in the next stage of Hawkins’ approach.

Developing a complex rehabilitation intervention during a period of unprecedented service pressures required ongoing reflexive attention to process as well as outcome. In line with calls for greater transparency about the “work” of co-design and the practical challenges of participatory intervention development [21, 46] we offer a series of reflections intended to support researchers undertaking similar endeavours. These reflections draw on our experience of delivering a ten-workshop co-design process during the COVID-19 pandemic and highlight key methodological, relational, and practical considerations.

The following subsections explore issues related to leadership and facilitation in online co-design, building psychological safety and rapport, communicating complex data across stakeholder groups, sustaining engagement through structured feedback loops, negotiating the role of families and informal carers, and adapting intervention development to the constraints of a global pandemic. Together, these reflections illustrate the dynamic, negotiated, and relational nature of co-design and aim to offer transferable learning for others developing complex interventions in rehabilitation contexts.

Power dynamics between professionals and public contributors are a recognised challenge in co-design, with the risk that clinical voices may dominate discussions and influence decision-making [47]. Effective workshop leadership is recognised as critical to steering complex co-production processes [48], as insufficient facilitation can lead to fragmentation, lack of direction, or inequitable participation [25]. The ethos of EBCD emphasises partnership and shared leadership [36]. Donetto et al. [46], highlight the importance of clinical leadership in sustaining project momentum. However, when researchers assume leadership roles, there is a risk of introducing power asymmetry that may undermine genuine collaboration [47].

In this study, leadership was deliberately enacted as a facilitative rather than directive function. The lead researcher acted as coordinator and enabler, setting agendas collaboratively, maintaining focus, and creating conditions for equitable contribution. Rousseau and colleagues [49] describe leadership as a stabilising factor in co-design, providing vision and coherence without imposing hierarchy. This approach was particularly important in an online environment, where subtle social cues and informal bonding are harder to achieve.

Two recent reviews found that imbalances in power dynamics were identified in co-design studies [48, 50]. Both reviews conclude that while genuinely equal decision-making may be idealistic, fostering more democratic processes and achieving a ‘parity of esteem’ among participants is both realistic and achievable. Our experience aligns with these findings: while the research team retained responsibility for ensuring methodological rigor and ethical compliance, the workshop structure, collaboratively agreed ground-rules and iterative feedback mechanisms supported shared ownership and collective decision-making.

Although efforts were made to support equitable participation, the stakeholder group included a greater number of healthcare professionals than stroke survivors and informal carers. This imbalance may have influenced the direction of discussions and prioritisation of intervention components, despite the facilitative strategies used to promote inclusive contribution and shared decision-making. The online format and communication demands of repeated workshops may also have created additional barriers for some stroke survivors, particularly those experiencing fatigue, aphasia, or reduced confidence in group discussion. These limitations highlight the ongoing challenges of achieving fully equitable co-design within complex rehabilitation research.

Bringing together clinicians, stroke survivors, and informal carers in the same workshop introduced challenges of language and expertise. Professional jargon and technical terminology risked alienating non-clinical participants. To address this, the group co-developed explicit ground rules based on co-production principles [28, 51] including “no jargon,” “equal voice,” and “ask if unclear.” These rules were revisited at the start of each workshop and adjusted collaboratively, ensuring that all participants had agency in maintaining inclusivity. Although clinicians occasionally reverted to professional shorthand, patient and carer stakeholders felt empowered to query unfamiliar terms. This mirrors findings from Broomfield [52] who created accessible definitions to bridge professional–lay communication gaps.

The workshops were conducted online due to COVID-19 restrictions. A well-documented challenge in digital participatory research is building rapport and trust when participants engage remotely [35]. Shamsuddin and colleagues [17] note that discussing sensitive experiences online can initially make participants feel detached; yet paradoxically, virtual environments can create a safer space for disclosure due to a greater sense of personal control and privacy. This was reflected in the BISTRo workshops. The introductory icebreaker, sharing personal food and drink stories, proved highly effective in establishing warmth and connection. Participants laughed, empathised, and quickly formed a cohesive group bond. Hall et al., and colleagues [28] suggests that workshops benefit from facilitation from a facilitator who is engaging and can motivate others to share ideas. The use of trigger videos further enhanced emotional connection [23]. By portraying authentic patient and staff experiences, the videos stimulated empathy, validated lived experience, and grounded subsequent discussions in shared understanding. These multimedia tools acted as effective anchors in an otherwise text-heavy online environment.

Maintaining engagement across ten sequential workshops required that the research leader undertake deliberate relational work [47]. The facilitation approach combined consistency (structured agendas, timekeeping) with flexibility (space for humour, storytelling, and digression). As a result, participants described the sessions as enjoyable, inclusive, and emotionally safe, key prerequisites for genuine co-production [50].

Translation also extended to the use of multiple data sources within the earlier part of the study (Stage 1 of Hawkins’ approach to intervention development) [22]. In the workshops, stakeholders were asked to interpret ethnographic observations, interview data, and workshop transcripts, which sometimes present conflicting perspectives. Rousseau and colleagues [49] caution that tacit knowledge from stakeholders, while invaluable, can overshadow empirical evidence if not balanced carefully. To manage this, the lead researcher synthesised data into visual mind maps and matrices that supported shared interpretation without privileging academic or professional voices. Thus, in the workshops, the communication strategy combined structural scaffolding (rules, visuals, summaries) with relational sensitivity (respectful dialogue, iterative checking). This dual focus fostered both comprehension and co-ownership of decisions [47].

Feedback loops and iterative reflection were central to maintaining transparency and momentum within and between the workshops [16]. Each workshop was audio-recorded, summarised, and shared within one week, alongside a “feedback pack” containing key discussion points, decisions, and review materials. This system enabled participants who could not attend live to contribute asynchronously via email or phone, reducing attrition and supporting inclusivity. Hall and colleagues [28] emphasise that workshop reflection and feedback are vital to stakeholder engagement, ensuring data credibility and participant satisfaction. Similarly, LeBlanc and Nosik [53] recommend using structured checklists to evaluate meeting effectiveness and participation equity. In BISTRo, post-session reflections were guided by a structured template noting whether all participants had access to materials, opportunities to speak, and clarity on next steps. This process not only improved documentation and rigor but also created a rhythm of accountability and continuity that sustained engagement across the four-month co-design period.

The potential role of family members in the breakfast group intervention was a recurrent topic of discussion in the workshops. Participants acknowledged that family involvement could enhance continuity of care and align with the 2023 NICE stroke rehabilitation guidelines [15], advocating for family participation in goal-setting and rehabilitation. However, during the COVID-19 period, visitor restrictions and concerns about patient comfort limited direct involvement of family members. As an alternative, the SIDG decided to involve families indirectly through the patient booklet, facilitating them to contribute personal information, preferences, and reflections at discharge. This flexible approach balanced infection control realities with the principle of person and family-centred care. Future studies should explore structured mechanisms for family inclusion when visiting restrictions are not in operation.

This study integrated a structured intervention development framework with Experience-Based Co-Design [19, 21], ensuring that the intervention was informed by both programme theory and lived experience. Creative co-design methods, including trigger videos and virtual whiteboards, supported emotional engagement and facilitated in-depth exploration of stakeholder perspectives during workshops. Methodological transparency was strengthened through iterative documentation processes, such as a considerations log and workshop feedback summaries, which provided clear traceability of co-produced design decisions.

Delivering workshops online increased geographical reach and offered flexibility for participants; however, this approach may have excluded individuals with limited digital access or confidence. Despite efforts to support inclusive participation, the requirement for participants to engage in online group discussion may have excluded some stroke survivors with more severe aphasia, cognitive impairment, fatigue, or reduced confidence using digital platforms. As a result, some perspectives may have been underrepresented within the co-design process. To mitigate this, workshop materials were circulated in advance using accessible formats, supported communication strategies were used throughout discussions, and visual tools such as mind maps, prompts, and aphasia-friendly resources were incorporated to facilitate participation. Communication-focused adaptations within the intervention were strongly shaped through collaborative discussions involving stroke survivors, carers, and speech and language therapists, alongside findings from earlier qualitative interviews and observational research. Training sessions were provided to partially mitigate this limitation. Representation from minority ethnic groups was limited, and there was an imbalance in numbers between healthcare professionals and public contributors, which may have influenced discussion dynamics during co-design.

### Implications and future directions

This study provides empirical and methodological contributions to the field of rehabilitation intervention development. First, it demonstrates that co-production can be achieved effectively online when supported by strong facilitation, iterative feedback, and clear communication scaffolds. Second, it illustrates how hybrid frameworks, combining Hawkins’ [16] systematic structure with EBCDs [19, 22] participatory ethos, can yield interventions that are both rigorous and relationally grounded.

The BISTRo model operationalises psychosocial rehabilitation by embedding eating and drinking within a socially meaningful, multidisciplinary context. It aligns with emerging priorities in stroke care [15, 54], emphasising identity reconstruction, peer interaction, and person-centred recovery [15].

Future research should focus on the later phases of the MRC guidance for developing and evaluating complex interventions [26]. The next step is to assess the intervention’s feasibility and acceptability across diverse settings, and if that is successful, measure the effectiveness and cost-effectiveness in a future evaluation.

## Conclusion

Co-production through stakeholder workshops generated a contextually grounded intervention for eating and drinking difficulties after stroke. Integrating Hawkins’ structured framework with Experience-Based Co-Design enabled a balance of theoretical rigor, empirical evidence, and lived experience. Despite the constraints of online delivery during the COVID-19 pandemic, the participatory approach fostered strong engagement and shared ownership among stakeholders.

The Breakfast Group Intervention for Stroke Rehabilitation (BISTRo) offers a novel multidisciplinary model that embeds therapeutic eating, social connection and psychological support within routine rehabilitation. It addresses psychosocial aspects of recovery that are often overlooked in traditional stroke care.

## Supplementary Information

Below is the link to the electronic supplementary material.

Supplementary Material 1

Supplementary Material 2

Supplementary Material 3

## Data Availability

The data sets supporting the conclusions of this article are available in the White Rose eTheses Online repository: https://etheses.whiterose.ac.uk/id/eprint/34199/Additional anonymised materials can be provided by the corresponding author on reasonable request. For the purpose of open access, the author has applied a Creative Commons Attribution (CC BY) licence to any Author Accepted Manuscript version arising.

## Abbreviations

BISTRo: Breakfast group intervention for stroke rehabilitation
CReDECI 2: Criteria for reporting the development and evaluation of complex interventions (version 2)
EBCD: Experience-based co-design
GRIPP2: Guidance for reporting involvement of patients and the public
GUIDED: GUIDance for the rEporting of intervention development
HRA: Health research authority
IRAS: Integrated research application system
MRC: Medical research council
NHS: National health service
NIHR: National institute for health and care research
PPI: Public and patient involvement
REC: Research ethics committee
SIDG: Stakeholder intervention development group
TIDieR: Template for intervention description and replication
