# Supplementary material for: Developing a rehabilitation intervention for eating and drinking difficulties following stroke through co-design stakeholder workshops
Source: BMC Health Serv Res. 2026 Jun 23;26:1248. doi: 10.1186/s12913-026-14977-2 (PMC13556001; doi:10.1186/s12913-026-14977-2)
Supplement: Supplementary file 1 [file 12913_2026_14977_MOESM1_ESM.docx]

**Supplementary Figures**

**Supplementary Figure 1: Environmental Layout a) Site 1 b) Site 3**

a) **Site 1 Layout**

**b) Site 2 Layout**

**Supplementary Figure 2: Food Preparation Station (Site 1)**

**Supplementary Figure 3: Food Preferences (Booklet illustration)**

**Supplementary Figure 4: Patient completing the daily log in the patient booklet.**

**Supplementary Figure 5: Healthy Eating Illustrations**

**Supplementary Figure 6: Codesign Poster (used in the open research event to engage key stakeholders).**

**Supplementary Figure 7: Stroke Survivor Poem**

**Supplementary Figure 8: Artefacts from the open research event a) Patient Booklet**

**b) Illustrations c) The Breakfast Group d) Food Preparation**

a) **Patient Booklet	b) Illustrations with quotes**

c)**The Breakfast Group	d) Food Preparation**
