# Supplementary material for: Developing a rehabilitation intervention for eating and drinking difficulties following stroke through co-design stakeholder workshops
Source: BMC Health Serv Res. 2026 Jun 23;26:1248. doi: 10.1186/s12913-026-14977-2 (PMC13556001; doi:10.1186/s12913-026-14977-2)
Supplement: Supplementary file 2 [file 12913_2026_14977_MOESM2_ESM.docx]

**Supplementary Information: Checklists**

**Supplementary Table 1. TIDieR Description of the BISTRo Interventio****n**

| Item | Description |
| --- | --- |
| 1. Brief Name | Breakfast Group Intervention for Stroke Rehabilitation (BISTRo). |
| 2. Why (Rationale) | BISTRo was developed as a structured rehabilitation intervention addressing the physical, social, and psychological consequences of eating and drinking difficulties after stroke. Although mealtime groups exist in stroke rehabilitation, current literature offers only limited and unstructured descriptions and lacks a multidisciplinary model. Guided by an intervention-development framework [16] , the multi-component intervention was co-designed with stakeholders. |
| 3. What (Materials) | Patient materials: A5 illustrated booklet including personal food/drink preferences, portion size, abilities and support needs, clinical outcome measures, treatment goals, daily log, and discharge advice. Staff materials: Staff intervention manual; training package; Standard Operating Procedure; risk-assessment template; pre-intervention assessment proforma; aphasia-friendly menus; communication stickers; template name plates; visual cue stickers; conversation-support resources; environmental setup checklist; "do not disturb" signage; first-up boards; attendance and "ready for breakfast" stickers; staff reflection log. |
| 4. Procedures | Pre-intervention assessment (physical function, social eating history, dietary preferences, stroke impact on eating/drinking abilities, required modifications, clinical measures). Patients were then invited to join the daily group. Sessions involved breakfast preparation and consumption, supported by staff assisting with posture, swallowing, mobility, communication, and social engagement. Patients walked or were assisted to the dining space set up café-style (tablecloths, flowers, name plates, napkins). Tables promoted independence (e.g., drinks to self-pour). Preparation stations allowed patients to make breakfast with supervision. Patients completed their booklet entries each session (daily log, reflections, plans for the next day). |
| 5. Who Provided | Delivered by multidisciplinary staff (occupational therapists, speech and language therapists, dietitians, physiotherapists, psychology assistants, nursing staff, and support workers) trained using the BISTRo manual and training materials. |
| 6. How  (Delivery Mode) | Online pre-intervention staff training. Face-to-face patient  sessions in dining rooms or adapted ward spaces. |
| 7. Where | Inpatient stroke rehabilitation wards across three NHS sites. |
| 8. When and How  Much | Daily sessions, five days per week, lasting 45–60 minutes. Patients participated until discharge; new patients joined as appropriate. |
| 9. Tailoring | Core components (standardised): Pre-intervention assessment; environmental setup checklist; patient booklet; group procedures. Adaptable components: Group size (based on staffing/patient acuity); local risk assessment; personalised treatment plans and goals; aphasia-friendly menu adaptations. |
| 10. Modifications | Adjusted for infection-control standards (distanced seating, PPE, enhanced cleaning, reduced shared items). Dedicated gluten-free preparation station. Simplified menus and communication aids for aphasia accessibility. |
| 11. Fidelity  Assessment | Environmental checklists and staff reflection logs are used to monitor adherence and record learning. |
| 12. Planned  Evaluation | A further feasibility study is planned for the future, followed by a multi-site clinical trial. |

**Supplementary Table 2. CReDECI 2 Checklist (Development Phase) for the BISTRo Intervention [31]**

| CReDECI 2 Item | Description of how the study met the criterion |
| --- | --- |
|  |  |
| 1. Description of the intervention’s underlying theoretical basis | The intervention was underpinned by rehabilitation theory, particularly principles of psychosocial recovery, identity, participation and therapeutic engagement in everyday activities. Hawkins’ intervention-development framework and Experience-Based Co-Design (EBCD) informed the development process by providing methodological structure and ensuring that lived experience shaped the design. A co-produced logic model articulated proposed mechanisms of action and intended outcomes. |
| 2. Description of all intervention components, reasons for their selection, and aims/essential functions | Components were identified through evidence synthesis (systematic review, ethnography, interviews) and workshops. Patient-facing materials (booklet, menus, communication tools), environmental setup procedures, multidisciplinary facilitation, and daily session routines were developed because they addressed needs identified by stakeholders (e.g., safety, autonomy, dignity, social connection). Essential functions included enabling safe eating, promoting independence, supporting psychological well-being, and enhancing participation. A full TIDieR description is provided in Table 1. |
| 3. Illustration of intended interactions between components | The logic model and workshop decisions demonstrated how components interact: environmental set-up enables independence; communication aids support safe food/drink choices; multidisciplinary facilitation integrates physical, cognitive, and social rehabilitation; and peer interaction reinforces psychosocial recovery. The group session is the point at which these elements operate synergistically. |
| 4. Description and consideration of contextual characteristics in intervention modelling | Contextual factors were explicitly considered, including stroke-ward workflows, staffing variability, COVID-19 infection-control policies, cultural variation in food practices, digital access challenges during online co-design, and inter-site operational differences (including a ward move). All prototype refinements across Sites 1–3 responded directly to contextual constraints and opportunities, ensuring the intervention was feasible, acceptable and locally adaptable. |

**Supplementary Table 3: The GRIPP-2 Reporting Checklist adapted from Staniszewska et al., [33]**

| **GRIPP2- Domain** | **How the BISTRo Study Addresses the Item** |
| --- | --- |
| 1. Aim of PPI | The purpose of involving stroke survivors, carers, and rehabilitation professionals was to co-design a contextually grounded intervention addressing eating and drinking difficulties after stroke. Stakeholder expertise was sought to ensure real-world feasibility, acceptability, and alignment with lived experience. |
| 2. Background to PPI activity | PPI was integral throughout all stages of intervention development. Stakeholders contributed insights into clinical routines, patient needs, cultural food practices, cognitive and communication barriers, and contextual constraints on stroke wards. |
| 3. PPI roles and responsibilities | A Stakeholder Intervention Development Group (SIDG) of 15 people (stroke survivors, carers, MDT staff) attended 10 workshops. All contributed to agenda-setting, reviewing materials, co-design decisions, prototype testing feedback, booklet design, environmental set-up, dietary communication tools, illustrations, and staff training content. |
| 4. PPI identification and recruitment | Participants were recruited through NHS clinical sites and stroke networks to achieve variation in lived experience, profession, role, and stroke severity. Inclusion considered diversity of dietary needs, communication profiles, and rehabilitation experiences. |
| 5. Nature and extent of PPI | PPI was continuous across 10 workshops, prototype testing in 3 sites, and iterative revision stages. Stroke survivors influenced environment design, booklet content, depiction of stroke in illustrations, dietary information, communication strategies, seating layout, ground rules, and safeguarding dignity. |
| 6. Training and support provided | Online workshop orientation, co-design principles, trigger video briefings, EBCD approach, ground rules, and accessible materials (aphasia-friendly slides, visuals) supported equitable participation. Facilitation emphasised shared voice, clarity, no jargon, and emotional safety. |
| 7. Methods used for PPI | Workshops included structured co-design exercises, trigger videos, lived-experience storytelling, small-group breakout deliberation, voting, reflective logs, and prototyping reviews. Iterative documentation (“considerations logs”, summary tables) ensured transparency and continuity. |
| 8. Communication and inclusivity strategies | Ground rules, no-jargon policy, accessible documents, visual supports, and ongoing clarification mechanisms ensured clarity. Co-developed menus, dietary flags, name plates, and booklet prompts supported equal voice. Stakeholder-led challenges to language, cultural representation, weight framing, and dignity informed final design. |
| 9. Study phases in which PPI occurred | PPI informed systematic review interpretation, ethnographic insight synthesis, logic model development, component identification, toolkit prototyping, prototype refinement after site testing, staff manual finalisation, and dissemination event design. |
| 10. Ethical considerations in PPI | Participants received study information, consented to recorded workshops, and could opt-out. Voucher recognition, emotional safeguarding, and respect for personal narratives were embedded. Data sharing logs ensured transparent documentation of decisions. |
| 11. Resources for PPI | Workshops were online, eliminating travel burden. Trigger videos, photos, lived-experience quotes, environmental checklists, draft booklets, and prototype feedback packs supported meaningful participation. External illustrator attended to incorporate stakeholder vision of inclusive and dignified representation. |
| 12. Governance and decision-making | Decisions were made through consensus dialogue, reflective review of prototype testing, and iterative logs documenting rationale. Workshop outputs were circulated for review between sessions. Stakeholders determined core vs. adaptable components for fidelity testing. |
| 13. PPI influence on intervention design | Direct PPI influence included: redesigned patient booklet (e.g., daily logs, sticker attendance, simplified goals); culturally inclusive illustrations; gluten-free prep station; infection-control layout; dietary communication tools; table set-up guidance; name plate dietary prompts; family input pages; removal of weight-recording section. |
| 14. PPI influence on research process | Stakeholders shaped workshop agendas, logic model wording (e.g., “mastery” reframed as “confidence and participation”), co-authored ground rules, and supported organisational negotiations during ward relocation and infection control changes. Their reflections informed feasibility considerations and dissemination planning. |
| 15. Outcomes and impact of PPI | Stakeholder perspectives ensured the intervention addressed psychosocial identity, dignity, pleasure, social connection, and meaningful recovery—not solely eating safety. Prototype testing in 3 sites confirmed feasibility and prompted refinements grounded in stakeholder insight. Engagement sustained across all workshops. |
| 16. Challenges and limitations of PPI | Online delivery risked digital exclusion; minority ethnic representation was limited; emotionally sensitive discussions required careful facilitation; power asymmetry needed ongoing management. COVID restrictions limited direct carer involvement at bedside. |
| 17. Critical reflection | Co-design enabled transparency and shared ownership but required sustained facilitation and emotional labour. Democratic processes, iterative logs, and reflective review mitigated power imbalance. Online methods proved inclusive and flexible despite initial concerns. Future cycles should deepen cultural outreach and explore structured family participation. |

**Supplementary Table 4: GUIDED Reporting Checklist based on Duncan et al 2020 [32]**

| **GUIDED Item** | **Checklist Prompt** | **How BISTRo Study Addresses the Item** |
| --- | --- | --- |
| 1 | Context of intervention development | Developed for UK NHS inpatient stroke rehabilitation wards. The study was undertaken during the COVID-19 pandemic. Context shaped delivery decisions, including infection-control adaptations and online workshop facilitation. |
| 2 | Purpose of intervention | To support eating and drinking after stroke by embedding therapeutic and psychosocial rehabilitation within daily mealtimes, targeting safety, independence, identity, confidence and social reconnection. |
| 3 | Target population | Adults receiving inpatient stroke rehabilitation with eating/drinking difficulties. Co-design included stroke survivors, family informal carers and multidisciplinary staff, with attention to aphasia, dietary needs and cognitive impairment. |
| 4 | Use of a published intervention development approach | Hawkins’ staged framework structured development; EBCD guided participatory co-production, use of narratives, equitable decision-making and iterative prototyping. |
| 5 | Evidence sources informing development | Systematic review, ethnographic fieldwork, interviews, observations, workshop dialogue, prototype testing across three wards and trigger films shaped component decisions. |
| 6 | Use of theory | Underpinned by rehabilitation theory addressing psychosocial recovery, identity reconstruction, participation, self-efficacy and therapeutic engagement in everyday tasks. A co-produced logic model mapped mechanisms to intended outcomes. |
| 7 | Incorporation of existing practice components (if applicable) | Existing informal breakfast practices informed environmental setup and meal routines; BISTRo formalised these into structured procedures with clear components and fidelity expectations. |
| 8 | Guiding principles for decision-making | Principles included dignity, independence, safety, inclusivity, cultural sensitivity, transparency, shared ownership and organisational alignment. Ground rules and iterative documentation operationalised these. |
| 9 | Stakeholder involvement | SIDG of stroke survivors, informal carers and clinicians shaped all stages: defining components, revising the prototype after site testing, influencing illustrations, communication tools and booklet content. |
| 10 | Changes made during development and the reasons | Iterative modifications included booklet redesign, training manual refinements, infection-control spacing, gluten-free preparation station, environmental checklist and communication aids, driven by prototype testing and contextual constraints. |
| 11 | Adaptations for specific subgroups | Aphasia-friendly menus, communication stickers, culturally diverse illustrations, dietary flags, visual prompts and family input pages ensured accessibility and cultural relevance. |
| 12 | Remaining uncertainties | Unclear optimal staffing ratios, cost/resource needs, group size, implementation feasibility across different services and required intervention dose. To be addressed in feasibility testing. |
| 13 | Full description via TIDieR | Complete TIDieR table provided with materials, procedures, delivery, tailoring, fidelity assessment and evaluation plans. |
| 14 | Open access reporting and transparency | Study planned for open-access publication with supplementary outputs including decision logs, prototype materials, logic model, trigger films and patient poem. |
