# Supplementary material for: Developing a rehabilitation intervention for eating and drinking difficulties following stroke through co-design stakeholder workshops
Source: BMC Health Serv Res. 2026 Jun 23;26:1248. doi: 10.1186/s12913-026-14977-2 (PMC13556001; doi:10.1186/s12913-026-14977-2)
Supplement: Supplementary file 3 [file 12913_2026_14977_MOESM3_ESM.docx]

**Supplementary Videos 1: Digital Materials (Videos and Media)**

- Introducing the BISTRo Study: <https://youtu.be/b4vGBdhDpLA?si=-TLZG_Hc1gjZDXOe>

- Trigger Video 1: Exploring the importance of eating and drinking after stroke: <https://youtu.be/xhdwlYLu1VU>

- Trigger Video 2: The impact of eating and drinking difficulties after stroke: <https://youtu.be/s9gPNyNJDjM>

- Trigger Video 3: What to consider if you are planning a breakfast group intervention: <https://youtu.be/UQZ_3NRXBCo>

- An Occupational Therapist talking about ‘what matters to you’: <https://youtu.be/mFHxb6dkqUQ>

- The story of developing a breakfast group intervention for stroke survivors: <https://youtu.be/xTvDX6bs4vQ>

- Experience-based codesign with stroke survivors & informal carers in stroke research: <https://youtu.be/mFHxb6dkqUQ>

- Systematic Review: Psychosocial impacts of eating and drinking difficulties after stroke: <https://youtu.be/yCUxd_4hU28>
